# Key factors in the acceptability of treatment as prevention (TasP) in Scotland: a qualitative study with communities affected by HIV

**DOI:** 10.1136/sextrans-2014-051711

**Published:** 2014-12-08

**Authors:** I Young, P Flowers, L M McDaid

**Affiliations:** 1 MRC/CSO Social and Public Health Sciences Unit, University of Glasgow, Glasgow, UK; 2 School of Health and Life Sciences, Glasgow Caledonian University, Glasgow, UK

**Keywords:** HIV, GAY MEN, PROPHYLAXIS, QUALITATIVE RESEARCH, PUBLIC HEALTH

## Abstract

**Objectives:**

There is a clear need to understand the factors that might prevent and/or facilitate the effective use of HIV treatment as prevention (TasP) at an individual level. This paper reports on findings from the first qualitative study in the UK exploring the acceptability of TasP among gay, bisexual and/or men who have sex with men (MSM) and migrant African communities in Scotland.

**Methods:**

We conducted seven exploratory focus group discussions (FGDs) with convenience samples of MSM (five FGDs, n=22) and mixed-gender African (two FGDs, n=11) participants. Of these, three FGDs were conducted with HIV-positive MSM (n=14) and one FGD with HIV-positive Africans (n=8). We then conducted 34 in-depth interviews (IDIs) with a purposive sample of MSM (n=20) and Africans (n=14, women=10). Half were HIV-positive (MSM, n=10; African, n=7). FGD and IDI data were analysed thematically drawing on predetermined and emergent themes.

**Results:**

We found that inequalities in HIV literacy could be a barrier to TasP, as could social constraints, such as criminalisation of transmission, increased risk of sexually transmitted infection and increased burden of treatment. We also identified psychological barriers such as perceptions of risk. However, relationships and shared decision making were identified as potential facilitators for TasP.

**Conclusions:**

Our results suggest that potential use and management of TasP may not be straightforward. It could be contingent on reducing inequalities in HIV literacy, minimising the perceived burden of treatment and other potential risks, and addressing the dynamics of existing and socially acceptable risk management strategies, especially in relation to long-term serodiscordant relationships.

## Background

The discovery that antiretrovirals (ARVs) can prevent onward sexual transmission of HIV has significant implications for HIV prevention. Growing evidence has demonstrated that an HIV-positive person taking ARVs with undetectable viraemia is highly unlikely to transmit HIV to an unprotected sexual partner.^1–3^ UK HIV-expert organisations have endorsed this position, stating that the absence of sexually transmitted infections (STIs) and regular adherence to ARVs, in addition to undetectable viraemia, is an effective method of preventing HIV transmission in vaginal sex and significantly reduces risk in anal sex.^4^ Evidence also suggests treatment as prevention (TasP) works at community level through reducing population-level viraemia.^5–7^ WHO guidelines advise treatment initiation at a CD4 count of 500^8^ while a number of cities (San Francisco, Vancouver) and countries (France) have introduced policies to encourage the immediate treatment of people diagnosed with HIV to reduce community viral load and new infections.^9^ ^10^ However, evidence from the UK suggests a limited impact on community-level effectiveness^11^ and current UK treatment guidelines advise early treatment decisions be made on an individual basis.^12^

The effectiveness of TasP at a population level relies upon maximising the number of HIV-positive people at each level of the treatment cascade: (i) diagnosed with HIV, (ii) currently in care, (iii) taking ARVs and (iv) suppressed viraemia.^13^ Although the population-level treatment cascade has received much attention, there has been limited consideration at an individual level as to whether potential recipients of TasP will find this prevention strategy acceptable.^14^ Our review found only three such studies. These studies identified limited awareness of TasP, as well as scepticism about its effectiveness to prevent HIV transmission. There is a clear need to understand the factors that might prevent and/or facilitate the effective use of TasP. Drawing on extensive international evidence of HIV prevention successes and failures, Kippax and Stephenson have argued that a *social public health* approach is needed to better facilitate the use of ARVs for HIV prevention.^15^ That is, there is a need to understand the social context in which TasP-related HIV prevention is implemented. As we have argued elsewhere, this means that understanding the acceptability of TasP requires moving beyond expressions of willingness or adherence to medication and identifying the broader social, cultural and structural factors that will affect potential uptake and sustained effectiveness of TasP.^14^ This paper reports on findings from the first qualitative study in the UK exploring the acceptability of TasP among communities most affected by HIV. We identify a range of social factors and constraints that could affect the uptake and use of TasP on an individual level.

## Methods

**Figure 1:**
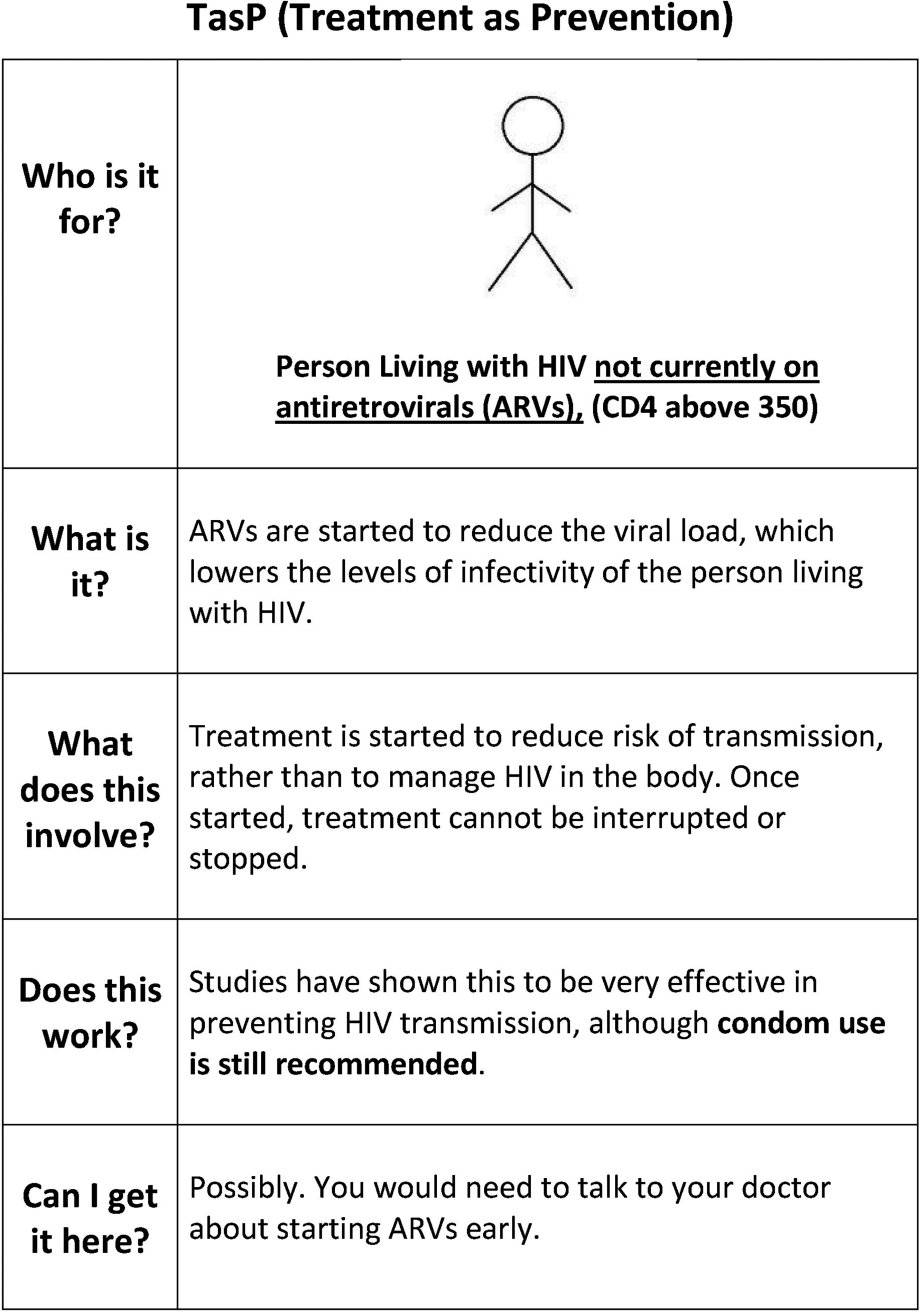
Treatment as prevention (TasP) card.

We employed mixed qualitative methods to explore the acceptability of TasP with the two communities most affected by HIV in Scotland: (1) gay, bisexual and/or men who have sex with men (MSM) and (2) men and women from migrant African communities. While Scotland is a low HIV-prevalence setting, these two communities represent over half of all new HIV diagnoses.^16^ We included HIV-positive and HIV-negative and/or untested participants in the study given the relational nature of sex and HIV prevention. First, we conducted seven exploratory focus group discussions (FGDs) with convenience samples of MSM (five FGDs, n=22) and mixed-gender African (two FGDs, n=11) participants aged 18–75 years recruited through community and/or support groups with the assistance of sexual health organisations in Glasgow, Edinburgh, Motherwell and Selkirk between August and November 2012. Of these, three FGDs were conducted with HIV-positive MSM (n=14) and one FGD with HIV-positive Africans (n=8). Participants were first asked about their understandings and management of sexual health risks, focusing on the role of sexual health technologies within these strategies. To facilitate discussion, participants were presented with a range of items, such as condoms, sachets of lubricant, a home pregnancy test, an emptied bottle of Truvada (ARV medication), a mock-up bottle of antibiotics, a list of ARVs available in the European Union, and images of an Oraquick^®^ In-Home HIV test and a rapid, fingerprick HIV test. In the second part, pre-exposure prophylaxis (PrEP) and TasP were explained to participants with the help of visual aids (figure 1). Participants were asked to discuss PrEP and TasP in relation to their own sexual health, including if and how they might be used, and to identify any barriers or facilitators to their use. Participants received £15 vouchers and travel costs at the end of FGD.

We then conducted 34 in-depth interviews (IDIs) between March and September 2013 to explore issues emerging from FGD findings and examine personal risk management practices in further depth. The purposive sample comprised MSM (n=20) and Africans (n=14, women=10) aged 19–60 years from Glasgow, Lothian, Lanarkshire and Grampian, half of whom had been diagnosed with HIV at the time of the interview (MSM, n=10; African, n=7). Recruitment was conducted via targeted flyers, posters and emails distributed through clinical, community and culturally specific non-sexual health avenues. Participants received £20 vouchers. Semistructured interviews explored PrEP and TasP acceptability within the context of existing risk management strategies. The first part of the interview focused on personal experiences of and perspectives on HIV, sexual health risk management practices and use of sexual health technologies. Participants were presented with a list of sexual health technologies that included all of the objects used in FGDs. The second part of the interview focused on the acceptability of PrEP and TasP, exploring awareness, potential use, concerns and combination with existing risk management strategies. The focus group and interview topic guides are available in the online supplementary material.

All FGDs and IDIs were digitally recorded and transcribed verbatim. Transcripts were anonymised and coded in NVivo 10. Data were analysed thematically, drawing on predetermined and emergent themes.^17^ ^18^ Rigour throughout the analysis was achieved through an iterative process of discussion with coauthors and revision of findings.^17–21^ In this paper, we present findings from the TasP-related discussions. Illustrative extracts referred to by number in the following section are provided in box 1.

Box 1 **Findings**

1. **Awareness and HIV literacy**
  - Limited awareness
    *he said that he had a low viral load and it was undetectable and…I remember googling it 'cause I was like 'what the hell does he mean?’ and, of course, because he said it was undetectable, I was thinking 'so that means that I could get it but then mine isn't detectable?’ So…I was thinking ‘I don't want…I don't want to put myself at risk and then I get an undetectable one and then they can't tell me I've got HIV because it's undetectable.’*  (HIV-negative MSM, IDI)
  - Diverse HIV literacy skills
    *Well, if they said ‘by taking your anti-viral medication’—and I do take it on time or anything like that, I'm good that way—[…]‘it makes your blood levels undetectable but it also makes your semen level undetectable.’ Then you'd be thinking differently. But they don't.*  (HIV-positive MSM, IDI)
2. **Social constraints and other risks**
  - Burden of treatment
    **R1:** The other point like the job of taking medication, [it] is not good…. It's frustrating ‘cause, it's not like side effects, it's just the idea of the work. I don't know, wake up in the morning have to take something it's…you can't have that freedom…I wake up, I just ok, you have to always getting them. The [partner] should agree with the condoms all the time…
    ***R2:** I just want to say from my experiences what I've seen that when people are faced with taking medication, it's that ‘do I take the medication now?’ is what we're saying, it might be taking it every day, every day is frustrating. Or do I wait until I'm ill to start taking it?*  (HIV-positive African FGD)
  - Other risks
    **R1:** If you're having unprotected sex without a condom, you're leaving yourself open to prosecution.
    **Q:** Even if your viral load is undetectable?
    **R1:** That's irrelevant.
    **R2:** Aye. You're still putting the guy at risk.
    **R1:** It's irrelevant, coz you're still, even though you're undetectable, you've still got HIV and you possibly could pass it on, so there's still […]
    **Q:** So actually, using a condom is about…legally protecting yourself? […]
    **R1:** …no, it's no just legally, but from what they've got as well, coz you don't know what they've got. It's no just yourself. Well, it's no just them you're protecting, it's yourself.
    **R2:** Mm hmm, coz you don't know their sexual health status, you know, if you don't ask, and then a lot of them will tell you lies, anyway.
    **R1:** And again, if they're already on any medication, the fact that you've got your viral load to come down to non-detectable, the last thing you want is to go into something where it goes back up again and you're having to re-fight the whole battle again.
    **R2:** Then, the thing is…, what is it, the medicine, like, see the tablets, like if you pick up a different strain.
    **R3:** Yeah, it can become resistant.
    ***R2:** It can become resistant to the tablets, as well, so there's a lot at stake, know what I mean?*  (HIV-positive MSM FGD)
3. **Perception of risk and treatment as prevention (TasP) candidacy**
  - High perception of risk, HIV-negative/untested
    *a bit risky* (HIV-negative African woman, IDI)
    *not safe* (HIV-negative African man, IDI)
    *Like Russian roulette* (HIV-negative MSM, IDI)
  - Low perception of risk, HIV-positive
    *I was getting a sort of spate of sort of infections then they said ‘maybe think about starting you on treatment to prevent other people from getting it’. I said no, it's because I was barebacking with sort of other positive guys I'm getting a lot more infections from them. I'm, whenever I was with negative guys I always used condoms so the risk of me passing it onto an HIV-negative guy's like very, very, very low because I use condoms…*  (HIV-positive MSM, IDI)
4. **Perceptions of treatment**
  - Social expectations to start treatment
    **R1:** If I was HIV and they gave me those drugs I would take them every day because…eventually you're gonna be taking drugs all the way through your life ‘cause you know it's helping you. So I don't think it would be a problem for them to be taking a drug that's helping them.
    **R2:** Some people know, they might get fed up of taking the drugs and hiding as well as you said there, they hide yeah? They can't go to anywhere they like without packing their drugs, they would be carrying their bag as if is baby, they can't leave their bag no, at some point and they don't see any changes. They might stop. Some people might get depressed and stop…
    ***R1:** …if people have a motivation, a reason for continuing to take the ARVs whether it's for good health, whether it's for keeping their partner safe. I think they are more likely to keep taking the ARVs…*  (HIV-negative African FGD)
  - Constrained choices
    *don't really have a choice because when we're pregnant we have to be on medication whether you like it or not.*  (HIV-positive African FGD)
5. **Relationships and shared decision making**
  - Initiating TasP as a prevention strategy with the right partner
    *I don't think I would start using it just for, like, anybody, I think, but if it was sort of a, someone, a very long term thing and, you know, we'd discussed everything.*  (HIV-positive MSM, IDI)
  - Supporting the sustainability of relationship
    *So perhaps if he'd known, or we'd known about, been told about those things, then it might have made it sort of, in your head sort of made you think, ‘right, this could go, you know, I would get what I want out of this long, relationship long-term.’*  (HIV-positive MSM, IDI)
  - Joint decision making
    *If you discuss it with your partner, and then if she says, ‘it's ok we can…we can not use condoms.’ Then that's fine. Yeah, that's fine, as long as you agree on what to do, the two of you.*  (HIV-positive African man, IDI)
  - Inequalities within relationships
    *It's up to him as well isn't it? To be convinced to say ‘yeah, it's safe now.’*  (HIV-positive African woman, IDI)
    *this guy I'm seeing now, you know, I'd like to have bareback sex wi’ him. But thinking how do I bring that issue up with him? And how would he…what would he think of me then? Would he be thinking 'are you fucking…you're willing to put my life at risk,’ you know? Because he wouldn't know anything about…I feel, I sometimes feel like saying to him ‘I've printed all this off for you, go and read it.’ But that's forcing somebody into something that if…if he felt like that…I think he knows that I would like to do that because of certain signs or something I've given him, but we've never discussed it.*  (HIV-positive MSM, IDI)

## Results

A range of factors that could affect the acceptability and uptake of TasP were identified and are described below.

### Awareness and HIV literacy

Lack of awareness and inequalities in HIV literacy were identified as barriers to TasP. Participants expressed very limited awareness of TasP as a ‘branded’ HIV prevention strategy. There appeared to be limited awareness of the link between suppressed viral loads and reduced levels of infectiousness. Awareness was affected by serostatus, with predominantly HIV-positive participants reporting knowledge of the link between an undetectable viral load and reduced HIV transmission. However, almost all of the participants, regardless of serostatus, appeared to struggle with the idea that someone living with HIV might *not* be infectious. For many, this doubt was grounded in the simple association of infectiousness with an HIV diagnosis, regardless of treatment status or viral load. Inequalities in HIV literacy skills among participants also affected TasP acceptability. For those, mostly HIV-negative participants unfamiliar with clinical terminology such as undetectable viral loads, TasP could be misunderstood and exacerbate stigma (1A). However, some of the HIV-positive participants described scepticism of TasP on the basis of their in-depth HIV knowledge (1B), highlighting how awareness and knowledge alone may not translate straightforwardly as acceptability.

### Social constraints and other risks

A number of social constraints were identified as barriers to TasP. HIV-positive participants, especially African men and women and MSM who had been living with HIV for a number of years, were critical of TasP as they expressed concerns about the burden of treatment, such as the daily work of taking ARVs (2A). HIV-positive participants also described other risks that might emerge as a result of TasP and/or that TasP would not address, such as the criminalisation of HIV transmission, the risk of STIs and the potential for developing resistance to treatment (2B).

### Perceptions of risk and TasP candidacy

Perception of risk was identified as an important psychological barrier to TasP candidacy. Our analysis showed clear differences in the ways that HIV-positive and HIV-negative participants considered risk in relation to TasP. HIV-negative participants commonly expressed an unwillingness to consider TasP as a prevention strategy in a serodiscordant sexual relationship due to a perception of *high* risk of HIV transmission (3A). Responses of many of these participants were generally framed by fear of any sexual contact with a known HIV-positive sexual partner.

For the remaining, largely HIV-positive participants, TasP was not embraced as a primary prevention strategy because of a *low* perception of risk of HIV transmission and a perceived lack of need for additional protection. Most described practising multiple methods of HIV prevention, including reliance on condoms, serosorting, avoiding ‘high-risk’ sexual acts or abstaining entirely from sex. These participants not only described their existing risk reduction strategies as effective, but also the extent to which they worked to maintain these efforts. As a result, most perceived risk of onward HIV transmission as minimal and were not immediately convinced of the need for additional protection. Moreover, all HIV-positive participants not taking ARVs expressed a desire to delay treatment initiation. One participant rejected TasP when it was offered to him in clinic because he was confident that risk of HIV transmission was minimal with his serodiscordant partners because he was already using condoms (3B). He further explained how his rejection of TasP was compounded by the perceived burden of treatment (eg, side effects, more easily identified as HIV-positive, increased insurance costs, worsened mental health). It appeared that for these HIV-positive participants, the *added* protection that TasP offered was neither necessary (as a result of existing risk-reduction strategies) nor a sufficient trade-off for their potential treatment burden. The latter could be avoided by delaying treatment initiation until deemed clinically necessary.

### Perceptions of treatment

Many HIV-negative participants described the perceived inevitability of taking ARVs and subsequent preventative benefits of treatment as a positive step for HIV prevention. While the broader benefits of good health and protecting a sexual partner contributed to this perspective, some HIV-negative participants identified social stigma as a potential barrier to TasP (4A). This FGD extract illustrates the social tensions between the imperative for people living with HIV to prevent onward transmission at any cost and the perceived experiences of HIV-related stigma and treatment burden. Moreover, many HIV-positive participants, especially African women, described a perception of constrained choice in relation to treatment initiation and the broader public health imperative of HIV prevention (4B).

### Relationships and shared decision making

The relational context of sex was identified as a potential facilitator to TasP among HIV-positive participants. Reliance on TasP without condoms was something that many HIV-positive participants said they would *only* consider in long-term serodiscordant relationships and with the ‘right’ partner (5A). While participants appeared cautious about the sexual partners with whom they would rely only on TasP, many were also optimistic about the possibilities TasP enabled, especially in terms of improved intimacy in long-term relationships. For example, one HIV-positive participant described how a previous serodiscordant relationship might have worked out differently if he had known about TasP (5B).

The decision to rely only on TasP within long-term sexual relationships was not a decision HIV-positive participants felt they could make alone, and many described how it would be a shared decision with a serodiscordant partner (5C). However, inequalities within relationships, especially in relation to gender and serostatus, were described by participants as complicating this shared decision making (5D). This was not a topic participants took lightly, and some described the difficulties in knowing if, how and when to raise the issue with a partner who may have limited HIV literacy (5E). Although shared decision making in sexual relationships was identified as a potential facilitator to TasP, many HIV-positive participants described feeling in a vulnerable position within serodiscordant sexual relationships. This meant potentially deferring TasP-related decisions to their sexual partners, if they were able to raise it at all.

## Discussion

This is the first study of TasP acceptability among communities affected by HIV in the UK. We found limited awareness of TasP, as noted elsewhere.^22^ ^23^ We identified a number of barriers and facilitators to uptake, and our results suggest that potential use and management of TasP may not be straightforward. It could be contingent on reducing inequalities in HIV literacy, minimising the perceived burden of treatment and other potential risks, and addressing the dynamics of existing and socially acceptable risk management strategies, especially in relation to long-term serodiscordant relationships.

Our findings in relation to TasP awareness and limited engagement with the contemporary science of HIV risk demonstrate that inequalities in HIV literacy could be a major barrier to TasP. While some HIV-positive participants demonstrated knowledge of clinical indicators of HIV risk, most participants expressed little understanding of and trust in the effect of non-detectable viraemia on risk of HIV transmission. This had implications not only for TasP but for identification of risk in relation to HIV more generally. Our findings echo Holt *et al*,^24^ who reported that Australian HIV-negative MSM were more sceptical than HIV-positive MSM of reduced risk of transmission with undetectable viral loads. Our findings suggest the need to address diverse levels of HIV literacy, especially in relation to the effective identification of and response to HIV risk based on contemporary clinical understandings of HIV, to ensure the appropriate understanding and uptake of TasP.

Our study identified a number of constraints to the effective and ethical use of TasP, especially in relation to additional risks for people living with HIV, such as risk of criminalisation of transmission or increased risk of STIs. While Kalichman *et al* reported an association between the belief that undetectable viral loads reduced HIV infectiousness and contracting a new STI in a US study with HIV-positive individuals,^25^ participants in our research were anxious about such risks. Moreover, participants expressed concerns about the burden of treatment that TasP might augment, for instance, through the additional work of adherence,^26^ poor mental health and the perceived constrained treatment choices resulting from the public health imperative to prevent HIV transmission. These findings demonstrate how TasP could increase the burden of *prevention* experienced by people diagnosed with HIV through the increased social, psychological and physical work in managing risk with ARVs. Although current HIV treatment guidelines in the UK state that the decision to start treatment must not be due to pressure from others,^12^ supporting people diagnosed with HIV in relation to TasP needs to consider the potential impact of these social burdens.

Interestingly, HIV-positive participants would only consider TasP and non-condom use with long-term serodiscordant partners. We suggest that potential non-condom use within this specific context should not be viewed as risk compensation,^28^ but as a carefully thought out risk reduction strategy within a trusted, monogamous relationship. In this way, relationships and shared decision making were identified as potential facilitators for TasP. Although participants did not always feel in a position to negotiate non-condom use, TasP did enable some to imagine improved longer-term sexual relationships. Consequently, TasP was seen in some cases as a prevention method with the potential for improved sexual health and well-being. These findings highlight the need to support shared decision making within relationships, while addressing potential inequalities, to achieve shared risk reduction through TasP.

### Limitations

With a small sample of MSM and migrant African participants from a low HIV prevalence setting, some of whom were engaged in sexual health or community services, our findings are not generalisable to a wider population but are transferable to similar populations in similar social contexts. For many in this study, TasP was a hypothetical concept and findings will be limited as a result of few direct experiences with TasP. However, our use of focus groups minimises this potential limitation as it addresses the social context and anticipated uptake of products.^27^

## Conclusion

The acceptability of TasP at an individual level will affect how effective it is as a population-level HIV prevention intervention. Our findings demonstrate that there is a need to improve HIV literacy and increase knowledge about TasP among those most affected by HIV so that informed and equitable treatment and risk reduction choices can be made. The recent Community Consensus Statement on TasP called for the safeguarding of the health and well-being of people living with HIV within the context of TasP,^28^ and our study highlights how effective implementation and support of TasP also needs to pay attention to the social and sexual context within which TasP will be used. Without this, we would suggest it has little chance of affecting on HIV prevention at the community level.

**Key messages**

- There is a clear need to understand the factors that might prevent and/or facilitate the effective use of treatment as prevention (TasP) at an individual level.
- Inequalities in HIV literacy, social constraints such as treatment burden and perceptions of risk were identified as potential barriers to TasP.
- Relationships and shared decision making were identified as potential facilitators to the effective use of TasP.
- TasP will be contingent on addressing inequalities in HIV literacy and other social constraints, and engaging with the dynamics of existing and socially acceptable risk management strategies.

## Supplementary Material

Web supplement
