# Supplementary material for: Key factors in the acceptability of treatment as prevention (TasP) in Scotland: a qualitative study with communities affected by HIV
Source: Sex Transm Infect. 2014 Dec 8;91(4):269–74. doi: 10.1136/sextrans-2014-051711 (PMC4453503; doi:10.1136/sextrans-2014-051711)
Supplement: Web supplement [file sextrans-2014-051711-s1.pdf]

Supplementary Material 1: Focus Group Discussion Guide

Part 1

Participants were presented with a number of objects to discuss. Objects included: condoms, sachets of lubricant, pregnancy test, list of ARVs, mocked up bottle of antibiotics, empty boxes/bottles of Truvada, and pictures of Oraquick home HIV test and rapid HIV test.

- **What do these objects make you think about?**
- **How do these objects relate to risk and HIV?**
- **What is risky in relation to HIV?**
- **Do you use these objects to manage HIV?**
- **What else do you use to manage HIV?**

Part 2

Provide visual cards of PrEP & TasP provided & explain separately.

- **How might you use these pills?**
- **How do you think your friends or sexual partners might use these pills?**
- **Are you concerned about the use of pills as a form of HIV prevention?**
- **Would these pills change the way people currently manage HIV? What do you think about this?**

Supplementary Material 2: Interview Topic Guide – HIV Positive Participants

1) Introducing experiences with HIV – their HIV 'story'

- When did you find out you were HIV positive? How did that happen?
- If/when did you start on ARVs/medication for HIV?
- If not on treatment:
    - How do you feel about not taking medication?
    - What were your expectations in relation to starting treatment?
- If on treatment – What have been your experiences of being on treatment?

2) Risk management/sexual health

- How does living with HIV affect how you manage your sexual health?
- What are the risks for you in relation to sexual health?
- You've said that you manage risk in sexual health by .... Has this changed and how?
- Do you talk to your sexual partners about this?
- Do you use health or other services to help you manage your sexual health? If so, how?

3) Use of existing technologies – *Use List*

List of sexual health technologies physically presented to participant:

condoms
HIV testing
Sexually transmitted infections (STI) testing
Contraception (the pill, IUD, long-term injections, etc)
pregnancy testing
CD4 counts
viral loads
Anti-retrovirals (ARVs)
post-exposure prophylaxis (PEP)

- Do you use any of these now? Have you used any of these in the past?
- How have you used them? What made you use them?
- Have you used any of these in combination with other prevention methods?
- How do you feel about using them?
- If you started/stopped using some of these, can you say why you did?

4) Potential use of new technologies

a) TasP

Approximate TasP description explained to participant:

Treatment as prevention is when ARVs are used by people living with HIV to not only to clinically manage HIV, but also to help prevent the transmission of HIV. TasP manages the 'viral load' or the amount of HIV in the system. Research has show that having an 'undetectable' viral load means that transmission of HIV is unlikely to happen. So, if the HIV positive person is taking their treatment regularly, and they don't have an STI, and their viral load is 'undetectable' for a period of time (about 6 months), they would clinically be considered not infectious. TasP in particular is when treatment is started to prevent transmission, rather than when the person clinically needs the treatment. If someone starts this treatment early – including for prevention reasons – they cannot stop taking this medication. So although people living with HIV will eventually move onto treatment, perhaps after 5 to even 10 years, this would mean starting treatment considerably earlier.

- Have you heard of TasP before?
- What do you think of TasP as a prevention method?
- How would you feel about using ARVs as prevention method?
- Can you imagine using this as a prevention method with a sexual partner?
- How do you think sexual partners would feel about using TasP as a prevention method?
- Do you have any concerns about this as a prevention method?
- Do you think other people living with HIV might use TasP as a method?

b) PrEP

Approximate PrEP description explained to participant:

Pre-exposure Prophylaxis (PrEP) is when ARVs are used by people who are HIV negative to prevent the transmission of HIV. At the moment, it can be taken once a day, although researchers are looking into other forms (short term PrEP, long-acting injectable, topical gel/microbicides). PrEP only works if people take the medication regularly. Clinical trials have shown that it is effective in relation to how often people take the pills. People are still encouraged to use condoms and other forms of risk reduction with PrEP use. There are some side effects, but this may not affect everyone and it generally seems to be well tolerated in the clinical trials. PrEP is not currently available in the UK, but it has been licensed for use in the United States.

- Have you heard of PrEP before?
- What do you think of PrEP as a prevention method?
- How would you feel about a sexual partner using PrEP as a prevention method?
- Do you have any concerns about PrEP as a prevention method?
- Do you think other people might use PrEP as a method?

Supplementary Material 3: Interview Topic Guide for HIV-negative or Untested Participants

1) **Experiences with and/or proximity to HIV**

- Is HIV a risk for you?
- Is HIV something that you talk about with your sexual partners? (or friends?)
- Have you ever tested for HIV?

2) **Risk management/sexual health**

- What are the risks for you in relation to sexual health?
- You've said that you manage risk in sexual health by ..... Has this changed and how?
- Do you talk to your sexual partners about how you manage your sexual health?
- Do you use health or other services to help you manage your sexual health? If so, how?

3) **Use of existing technologies** –

List of sexual health technologies physically presented to participant:

condoms<br>HIV testing<br>Sexually transmitted infections (STI) testing<br>Contraception (the pill, IUD, long-term injections, etc)<br>pregnancy testing<br>CD4 counts<br>viral loads<br>Anti-retrovirals (ARVs)<br>post-exposure prophylaxis (PEP)

- Do you use any of these now? Have you used any of these in the past?
- How have you used them? What made you use them?
- Have you used any of these in combination with other prevention methods?
- How do you feel about using them?
- If you started/stopped using some of these, can you say why you did?

4) **Potential use of new technologies**

a) PrEP

Approximate PrEP description explained to participant:

Pre-exposure Prophylaxis (PrEP) is when ARVs are used by people who are HIV negative to prevent the transmission of HIV. At the moment, it can be taken once a day, although researchers are looking into other forms (short term PrEP, long-acting injectable, topical gel/microbicides). PrEP only works if people take the medication regularly. Clinical trials have shown that it is effective in relation to how often people take the pills. People are still encouraged to use condoms and other forms of risk reduction with PrEP use. There are some side effects, but this may not affect everyone and it generally seems to be well tolerated in the clinical trials. PrEP is not currently available in the UK, but it has been licensed for use in the

United States.

- Have you heard of PrEP before?
- What do you think of PrEP as a prevention method?
- How would you feel about using PrEP as a prevention method?
- If you would use PrEP, how do you think you would you use it?
- Do you have any concerns about PrEP as a prevention method?
- Do you think other people might use PrEP as a method?

b) TasP

Approximate TasP description explained to participant:

Treatment as prevention is when ARVs are used by people living with HIV to not only to clinically manage HIV, but also to help prevent the transmission of HIV. TasP manages the 'viral load' or the amount of HIV in the system. Research has show that having an 'undetectable' viral load means that transmission of HIV is unlikely to happen. So, if the HIV positive person is taking their treatment regularly, and they don't have an STI, and their viral load is 'undetectable' for a period of time (about 6 months), they would clinically be considered not infectious. TasP in particular is when treatment is started to prevent transmission, rather than when the person clinically needs the treatment. If someone starts this treatment early – including for prevention reasons – they cannot stop taking this medication. So although people living with HIV will eventually move onto treatment, perhaps after 5 to even 10 years, this would mean starting treatment considerably earlier.

- Have you heard of TasP before?
- What do you think of TasP as a prevention method?
- Can you imagine using this as a prevention method with a sexual partner who is HIV positive?
- How would you feel if a sexual partner suggested this as an HIV prevention method?
- Do you have any concerns about this as a prevention method?
- How do you think other people who are HIV negative or untested might feel about using ARVs or HIV treatment as a prevention method?
